# Estimation and usefulness of measurement uncertainty from sampling at different spatial scales: microns to kilometres

**DOI:** 10.1007/s10653-024-01888-6

**Published:** 2024-03-13

**Authors:** Michael H. Ramsey

**Affiliations:** School of Life Sciences, University of Sussex, Brighton, BN1 9QG UK

**Keywords:** Measurement uncertainty, Sampling, Heterogeneity, Geochemical mapping

## Abstract

Uncertainty of measurement values (MU) is crucial to their reliable geochemical interpretation. MU can be estimated using the Duplicate Method, which requires the taking of a small proportion of duplicated samples, and can be applied at any spatial scale. The distance between the duplicated samples is selected to reflect the effect of analyte heterogeneity on the measurement result (i.e. estimated concentration) within each sampling target, at the particular scale of investigation. Three published case studies, at different spatial scales, are used to explain how the Duplicate Method can be applied to estimate MU. They also illustrate how MU can be used to improve geochemical interpretation and validate measurement procedures (that include sampling) by judging their fitness for purpose. At the kilometre scale, measurements from the GEMAS survey of agricultural soils across Europe are used to estimate their MU for the first time. The MU for 53 elements range from an uncertainty factor of 1.01 to over 10. The MU contributes more that 20% to the total variance for 8 of the 53 elements, showing that the measurement procedure was not fit for purpose in those cases. At the micron scale, measurements of oxygen isotopes in candidate quartz reference materials had MU that was dominated by its sampling component, caused by sometimes unacceptable heterogeneity. A third case study of Pb in soils at 12 UK sites showed that the Duplicate Method can also be used to quantify the heterogeneity (as factor 1.03 to 2.4), and that it can indicate different possible sources of an element.

## Measurement uncertainty in geochemistry

Data quality in geochemistry is traditionally expressed as an assessment of whether the precision and bias of analytical methods applied to the test samples are within acceptable limits. This assessment is seen as a necessary precondition to the subsequent interpretation of geochemical measurements. Where the data quality criteria were met, the subsequent geochemical interpretation then assumes that the measurement values are effectively ‘true values’ of the analyte concentration. The concept of MU was introduced in the 1990s and focussed on the quality of the measurement values, rather than of the analytical methods. Historically, MU was defined as ‘an estimate attached to a test results (*x*), which characterises the range of values within which the true value is asserted to lie’ (ISO, 1993). A value of MU (e.g. expressed as U) is attached to each ‘measurement value’ ‘*x*’, within a ‘measurement result’ *x* ± U (JCGM200, 2008) and summarises the effects of all of the possible causes of limited data quality, such as precision and bias, but also includes the effects of the other performance characteristics of analytical methods, such as selectivity, sensitivity, detection limit, working range, ruggedness. Crucially, the value of MU within each measurement result allows any user of the result to allow for the MU *during* the geochemical interpretation of the measurement value, not as a pre-condition *before* the making of an interpretation that then ignores MU.

A more recent definition of MU is ‘parameter, associated with the result of a measurement, that characterises the dispersion of the values that could reasonably be attributed to the measurand’(JCGM 100, 2008). In this case the words ‘true value’ are effectively equivalent to the ‘value of the measurand’, where measurand is the ‘quantity intended to be measured’(JCGM 200, 2008a). Although the words in the definition of MU have changed, the concept of MU is essentially the same.

## The role of sampling within the measurement process and within MU

Most of the effort on assessing and improving the quality of geochemical measurement values initially focused on the activity within chemical laboratories. However, it was gradually realised that the act of taking the primary sample also had a potentially large effect on the reliability of the geochemical interpretation of the measurement values. Initial attempt to improve sampling quality focused on designing and implementing ‘correct’ sampling procedures/plans (Gy, 1979). It was suggested that if a sample was taken ‘correctly’, by a ‘correct’ sampling protocol, then the sample would automatically be ‘representative’. By that assumption, the only remaining MU would be that arising in the chemical laboratory. The first approach to quantify the effect of sampling on the reliability of the subsequent geochemical interpretation was made by Miesch (1964) and further developed by Garrett and Goss (1980). Miesch suggested the taking of duplicate samples at a small proportion (e.g. 10%) of the locations used for geochemical mapping, with both of these samples also subject to duplicated chemical analysis in a ‘balanced design’. Miesch recommended the application of the statistical technique of analysis of variance (ANOVA) to all of these duplicated measurement values, to estimate the variance arising from both stages of the measurement process (i.e. sampling and analysis). What we would now call the ‘fitness for purpose’ (FnFP) of the geochemical measurements was then judged by comparing the variance from the sampling and analysis separately against that between the measured values of the analyte concentration at all of the locations used for duplicated sampling in the geochemical mapping (see the statistical model discussed below). This approach was based solely on analytical and sampling precision (and excluded the effects of bias and the other interlaboratory/sampler effects that are now included within MU). The traditional procedure to allow for imperfect data quality was therefore to assess the analytical data quality separately (rejecting it if it was over the upper limits for analytical precision and/or bias) and only to considered sampling precision as part of that FnFP criterion (via ANOVA). At that time sampling was not seen at part of the measurement process, and its effect was therefore not included in the precision or uncertainty of the analytical results.

This traditional approach was developed long before the concept of MU had been described or accepted. However, since MU has been generally accepted, it has now become more usual to include within it the contribution from the sampling procedure (UfS). The true value to be estimated (or value of the measurand) is therefore defined for a particular ‘sampling target’, which is defined as a ‘portion of material, at a particular time, that the sample is intended to represent’ (Ramsey et al., 2019), and is typically a batch of material or an area of land.

The estimation of MU (including UfS) is usually estimated using the Duplicate Method (described below) which utilises the same experimental design developed by Miesch, or some variant of that design. It is therefore now possible to improve the rigour of geochemical investigations in two ways. Firstly, it becomes possible to take an integrated view of the whole measurement process (i.e. sampling + analysis). Secondly, the MU value quoted within each measurement result (*x* ± *U*) includes a data quality summary for that individual measurement value (*x*) that can be used in any type of geochemical interpretation (i.e. not just in mapping).

This paper aims to show how sampling is part of the measurement process, and that MU should therefore include the contribution from the sampling procedure (i.e. UfS). Once a realistic value of the MU is known, it should be included in every measurement result that is reported to the person who is making the geochemical interpretation. At the validation stage, the value of the MU can be used to judge the FnFP of the whole measurement process and hence enable its quantitative validation. In routine operation, the MU within each measurement result (*x* ± *U*) can be propagated through each step of the geochemical interpretation in order to make decisions more reliable (e.g. probabilistic). Two examples will be used to demonstrate how MU (including UfS) can be estimated, and applied to interpretation, at any spatial scale, including both μm and km. At the km scale, the aim is to estimate the explicit MU for each measurement value (e.g. for agricultural soils across Europe) and consider its usefulness in geochemical interpretation. A third example will be used to demonstrate how the heterogeneity of the analyte concentration in the sampling target, which is often the dominant cause of UfS, can also be estimated by the Duplicate Method and used as an extra tool for geochemical interpretation at a range of spatial scales.

## Statistical model used for empirical MU estimation (including UfS)

The definition of MU from 1993 has the advantage that it includes the concept of ‘true value’, which can also be used to define the statistical model that explains the relationship between the *measured* value (*x*) and the *true* value (*X*_true_) of the analyte concentration in any one sampling target. The difference between these two values arises from the effects of both sampling and analysis on the measured concentration value, expressed as $\varepsilon_{{{\text{sampling}}}} + \varepsilon_{{{\text{analytical}}}}$. This relationship can be extended to include the effects of including multiple sampling targets that are encountered in practice and are also needed for reliable MU estimation (Ramsey et al., 2019), giving:

$$x={X}_{{\text{true}}}+{\varepsilon }_{{\text{between}}-{\text{target}}}+{\varepsilon }_{{\text{sampling}}}+{\varepsilon }_{{\text{analytical}}}$$

where ${\varepsilon }_{{\text{between}}-{\text{target}}}$ represents the variation of concentration between different sampling targets.

When we consider the estimated variances (*s*^*2*^) association with each of these terms.

we have

1 $${s}_{{\text{total}}}^{2}={s}_{{\text{between}}-{\text{target}}}^{2}+{s}_{{\text{sampling}}}^{2}+{s}_{{\text{analytical}}}^{2}$$

where ${s}_{{\text{sampling}}}^{2}$ is the between-sample variance on one target (largely due to analyte heterogeneity within the sampling target), ${s}_{{\text{analytical}}}^{2}$ is the between-analysis variance in one sample (usually expressed as repeatability), and ${s}_{{\text{between}}-{\text{target}}}^{2}$ is the variance between the multiple sampling targets. Because the measurement variance is defined as the sum of the sampling and analytical variance, this equation can be simplified as

2 $${s}_{{\text{total}}}^{2}={s}_{{\text{between}}-{\text{target}}}^{2}+{s}_{{\text{measurement}}}^{2}$$

This approach is generally effective, but does not include the extra variance that arises from the sampling bias of an individual sampler. This can be included by employing measurement values from multiple samplers on the same sampling targets, using, for example, data from either Sampling Proficiency Tests (SPT) or Collaborative Trials in Sampling (CTS). When ANOVA is applied to these results it is possible to also include s^2^_between-sampler_, which quantifies the between-sampler bias, within s^2^_sampling_ and hence also within ${s}_{{\text{measurement}}}^{2}$ and therefore within an estimate of MU (Ramsey et al., 2011).

## Ways of expressing and reporting MU

MU can generally be expressed in several ways, which are the standard uncertainty (*u,* typically ${s}_{{\text{measurement}}}$), the expanded uncertainty (*U* = *ku*, where k is usually 2 for 95% confidence), the expanded relative uncertainty (*U’*, relative to the concentration value, *x*) or the uncertainty factor (^*F*^*U*)(Ramsey & Ellison, 2015). Perhaps the simplest expression of MU is to quote the uncertainty interval (or confidence interval CI) between a stated lower confidence limit (LCL) and upper confidence limit (UCL). The LCL can be calculated, for example, as either *x*—*U*, or *x* /^*F*^*U*, and the UCL as either *x* + *U*, or *x* x^*F*^*U.* Another little-used alternative approach, relevant to this discussion, is to express MU as the proportion of the measurement variance within the total variance, as described in Eq. 2. MU expressed in proportional terms (e.g. as *U’*) can be very useful generally. However, this approach breaks down near the detection limit, where MU expressed as an absolute number (e.g. as *U*), if reliably estimated, is more dependable for the interpretation of a measurement value.

## Methods: estimation of MU (including UfS) using the duplicate method

The Duplicate Method is the most frequently used approach taken to estimate MU that includes UfS, at least its random repeatability components (Ramsey et al., 2019). Duplication is the most cost-effective form of replication, because it allows the maximum number (are therefore greatest diversity) of sampling targets to have duplicated samples taken from them. The taking of a triplicate sample, rather than a duplicate, will reduce the confidence interval (CI) on an estimate of MU at one particular sampling target (Roston et al., 2020). However, generally a better estimate of the typical MU over a whole geochemical survey (with a lower CI) is achieved by taking duplicate samples on more sampling targets than is possible with triplicate samples on fewer sampling targets. For the same budget, duplicate samples could be taken and 50% more sampling targets than are possible for triplicate samples. In special cases where there are very few (even just one) sampling targets, such as in forensic science, the higher levels of replication are justified to reduce the CI on the MU estimate.

The Duplicate Method only requires the participation of one ‘sampler’ (or measurement scientist). However, the estimate of MU can be made more realistic by including both between-laboratory reproducibility, and, as already noted, between-sampler bias quantified using multiple ‘samplers’, using results from either a CTS or a SPT (Ramsey et al., 2019, Sect. 9.4.1).

**Fig. 1:**
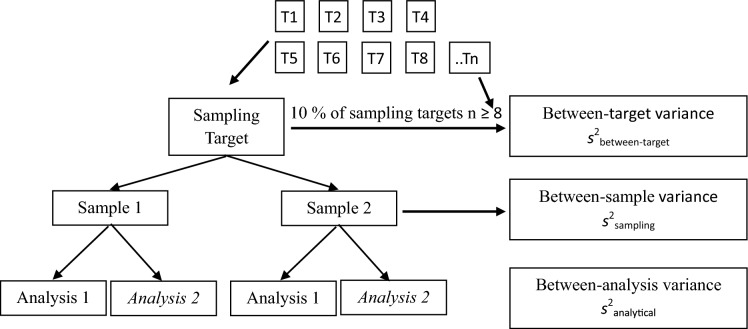
Full ‘balance design’ used for estimation of MU (including UfS) using the Duplicate Method, showing where the three component variances in Eq. 1 are estimated. In a variant called the ‘unbalanced design’, the second chemical analysis (*Analysis 2*) on either Sample 1 or Sample 2 can be omitted to save cost. In a second variant, called the ‘simplified design’, no duplicated chemical analysis are made (i.e. no *Analysis 2*), and an external estimate is used for the analytical component of MU (Adapted from Ramsey et al., 2019)

The Duplicate Method often uses a fully balance two-stage nested experimental design (Fig. 1) that requires the taking of duplicate samples, both with duplicate chemical analyses, on around 5 – 10%, but at least 8, of the sampling targets. An unbalanced experimental design is also possible to reduce costs (Rostron & Ramsey, 2012), but this results in a larger confidence interval on the resulting estimates of MU.

The taking of the duplicate samples in a realistic way is crucial and should never be done just by the splitting of single samples. Duplicate samples need to be taken independently by a fresh interpretation of the sampling procedure. The distance between where the duplicate sample are taken (in space or time) needs to reflect both the ambiguity in sampling procedure and the spatial (or temporal) uncertainty of the surveying device used. In this way the difference between the sample duplicates mainly reflects the effects of the analyte heterogeneity at that particular scale. These issues are best explained by use of an example, such as that discussed below.

## Estimation of MU at the macro (km) scale, using the GEMAS geochemical mapping of Europe data

**Fig. 2:**
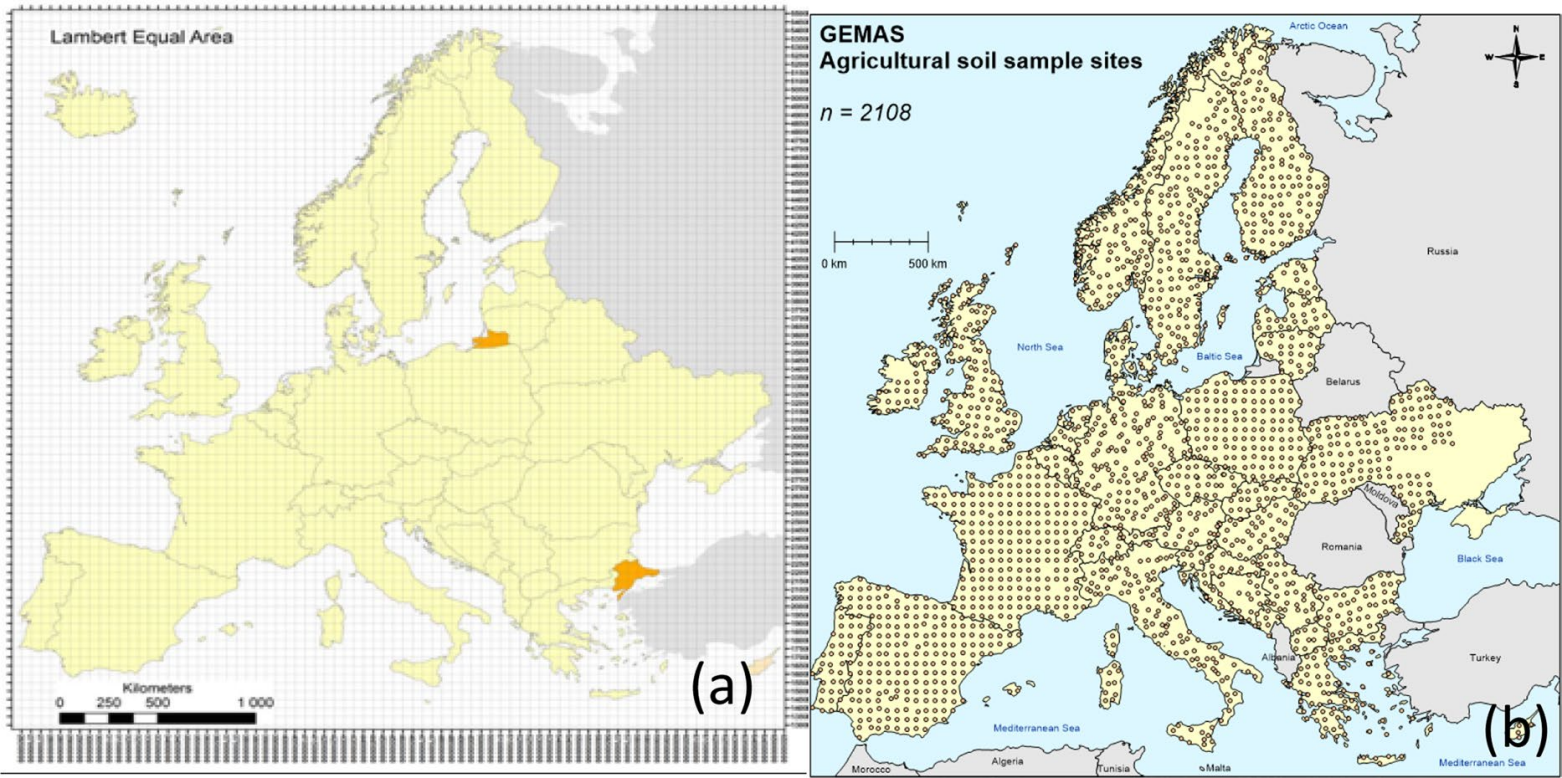
(a) Sampling grid of 50 × 50km squares (Reimann et al., 2014a, Fig. 3.1, p.32.) and (b) sample locations for agricultural soil for the GEMAS geochemical mapping (Demetriades et al., 2021, Fig. 1, p.22. Reproduced with permission)

The EuroGeoSurveys geochemical mapping of agricultural and grazing land soils project (GEMAS) sampled, amongst other media, the top soil (0-20cm) used for agricultural purposes (Reimann et al., 2009). A sampling grid of 50 km squares was constructed over Europe (Fig. 2a), and 2218 sampling locations (i.e. targets) were selected within each of the 2500 km^2^ grid squares (Fig. 2b).

**Fig. 3:**
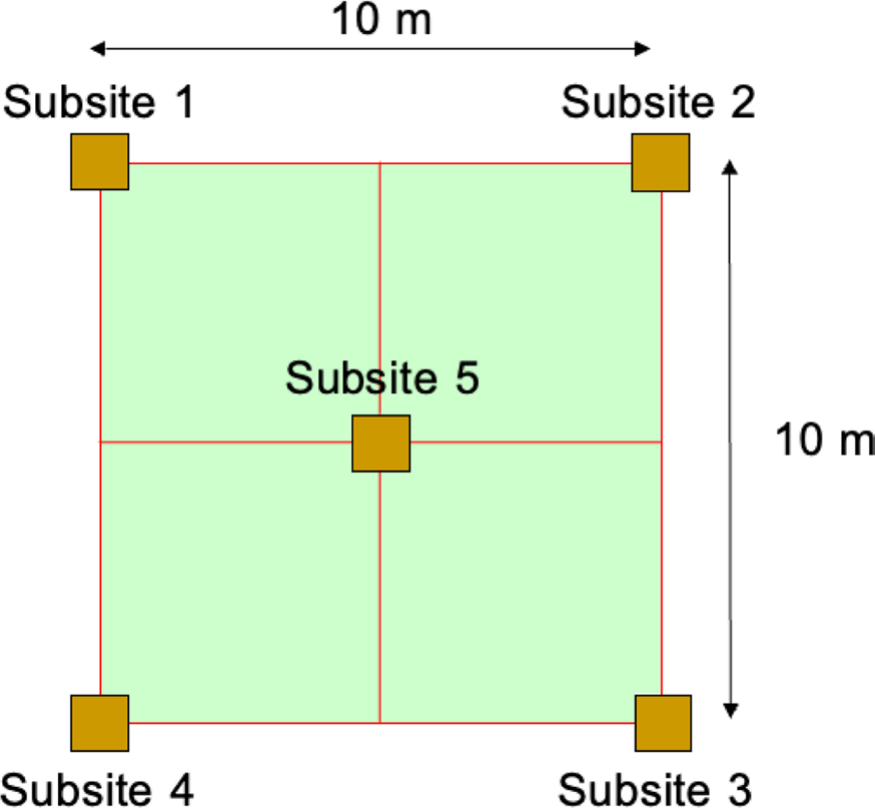
Experimental design for fivefold composite primary agricultural top soil sample (Reimann, 2014, Fig. 3.2, p.33. Reproduced with permission.)

Each sampling target was typically one large arable field (meadow) with dimensions greater than 25 × 50 m that was selected as being away from visible sources of contamination and therefore giving the ‘most representative’ sample for each cell (Reimann, 2014). Within each sampling target a fivefold composite sample (of mass 2—2.5 kg) was taken with a spade, over the depth of 0—20 cm, within a 10m square, using the design in Fig. 3.

A small proportion of the measurement values were used to decide on Fitness-for-Purpose (FnFP), using what was effectively the Duplicate Method, but not for the estimation of MU (Reimann et al., 2009). This employed an unbalanced design (Fig. 1, variant 1, with no second analysis (A2) on the first sample (S1)), followed by ANOVA. The FnFP criterion used for geochemical mapping was that the sum of the sampling and analytical variances (i.e. effectively the MU expressed as variance) should not exceed 20% of total variance (Ramsey et al., 1992), although this was not expressed in terms of the either the measurement variance or MU.

The final GEMAS data quality reports (Reimann et al., 2009, 2011) did not include any explicit MU values as mg kg^−1^ or %, but only implied as the % of total variance due to either sampling or analysis individually, but not their sum. One aim of the GEMAS study was to reveal the real geochemical variation of each element across Europe in a way that was not obscured by sampling and analytical variance (i.e. MU). The aim here is to estimate MU for the GEMAS measurement values explicitly, as either *U’* or as ^*F*^*U,* and consider its usefulness in geochemical interpretation, such as mapping.

For the assessment of FnFP, 104 duplicate composite agricultural soil samples were collected at ~ 5% of the 2218 sampling targets. The same sampling procedures were followed to collect the duplicate sample (S2) in the same field as for the original sample (S1), but at a different sub-site which has been estimated as approximately 10–20 m away (Demetriades, 2023).

All of the primary samples were centrally prepared (air dried, sieved to < 2 mm, homogenised and split into sub-samples) and randomized. They were then analysed, using duplicate sub-samples in the unbalanced design, for 53 elements determined by either ICP-MS or ICP-AES, after an Aqua Regia digest. Some of the elements (41) were also determined by XRF for comparison. In all cases the analytical bias was estimated using in-house reference materials (RMs), but not using the certified RMs that would have been preferable, and which would also have given better metrological traceability for the measurement results.

## Results: MU estimates for As by ICP-MS within GEMAS

The MU estimation procedures are best explained by initially considering the determination of one element, in this case As. When As was determined by ICP-MS, the analytical bias was estimated as approximately −1 mg kg^−1^, which can be considered negligible. In cases where there is an estimate of analytical bias which is statistically significant different from zero, it (and its own uncertainty) can be added into the estimated MU (Ramsey et al., 2019, p50).

A visual inspection of the measurement values for As arising from the unbalanced design (Table 1) shows quite good agreement of around 10% between analytical duplicates (S2A1 and S2A2). The duplicated samples (e.g. S1A1 and S2A1) show a less good agreement of around 20–30%.

**Table 1:** Measurement values for As by ICP-MS on the duplicated GEMAS agricultural top soils for the first 12 of 104 sampling targets that were duplicated using an unbalanced experimental design (Sample 1, Analysis 1 (S1A1), then Sample 2, Analyses 1 & 2 (S2A1 and S2A2))

S1A1	S2A1	S2A2
2.907	2.800	2.699
1.611	1.611	1.507
6.343	6.913	6.863
3.430	3.480	3.486
7.127	6.931	6.816
1.597	1.386	1.603
0.517	0.412	0.440
1.322	1.315	1.232
9.556	7.107	5.701
3.903	3.777	3.930
1.952	2.101	1.818
27.482	24.418	24.452

These initial visual observations can be quantified by applying ANOVA, but the measurement values must all be unrounded and untruncated. Unfortunately, this situation was not the case for As values for these same GEMAS soils when measured by XRF, where many of the lower concentration values are all reported as 1.5 mg kg^−1^ which is half of the estimated detection limit of 3 mg kg^−1^(Reimann et al., 2011).

The selection of the most appropriate type of ANOVA depends on the frequency distribution of the measurement values.

**Fig. 4:**
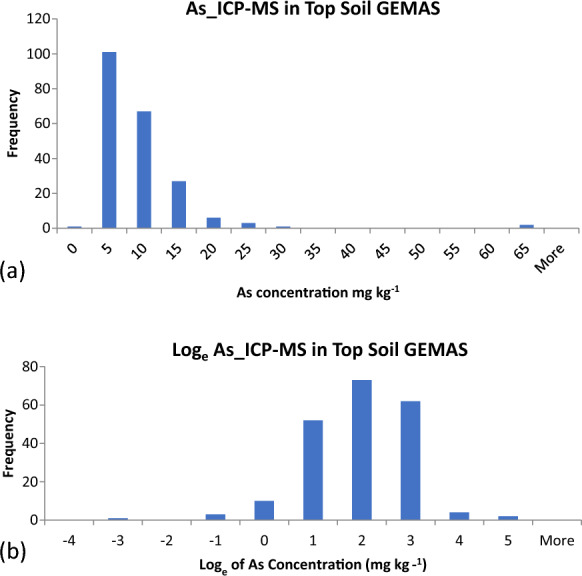
Histograms of frequency distribution for As in duplicated agricultural top soils in the GEMAS survey as **a** raw As concentration measurements, showing strong positive skew, and **b** log_e_-transformed measurements that show a distribution much closer to being normal (i.e. Gaussian)

The frequency distribution of the 312 (i.e. 104 × 3) As measurement values from the unbalanced design (Fig. 4a) is clearly not Normal (i.e. Gaussian), but positive skewed with many more lower, and fewer higher, values. The log_e_-transformation of these measurement values makes the distribution much closer to Normal (Fig. 4b), suggesting that the original distribution was log-normal. The software selected to apply ANOVA was RANOVA3 (AMC, 2023), which has options for (1) classical ANOVA (assuming Normal distribution), (2) robust ANOVA (assuming a Normal distribution with less than 10% outliers) and (3) classical ANOVA after automatic log_e_-transformation (assuming a log-normal distribution) to provide MU as the uncertainty factor.

**Fig. 5:**
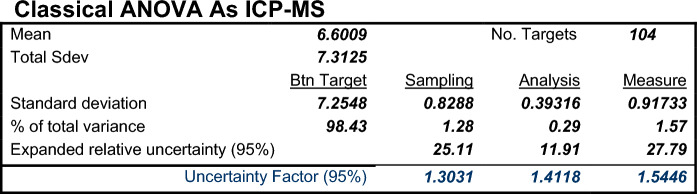
Output from RANOVA3 showing estimates of MU and its components in sampling and analysis, as both expanded relative uncertainty (line 6) and the uncertainty factor (line 7), and other statistics, for As measurements by ICP-MS in agricultural top soils from the GEMAS survey of Europe

The output of RANOVA3 for the 312 measurement values for As, in the format shown in Table 1, is given in Fig. 5.

The MU expressed as *U'* is 28% (27.79 on line 6 of Fig. 5), but this is *not* applicable as the frequency distribution is log-normal, not normal (Fig. 4). The MU is, therefore, better expressed as the Uncertainty Factor (^*F*^*U*) = 1.54 (1.5446, line 7 of Fig. 5). This output also shows that the MU is dominated by *U’*_samp_ with 82% of the measurement variance (i.e. 1.28/1.57%, on line 5 of Fig. 5), and *U’*_anal_ only contributes 18% (0.29/1.57%). Overall, MU contributes 1.6% (1.57%) of the total variance, which is less than the 20% that is recognised as the FnFP criterion for Geochemical Mapping (discussed below). This measurement procedure (sampling + analysis) is therefore FFP for this first element considered, As.

**Fig. 6:**
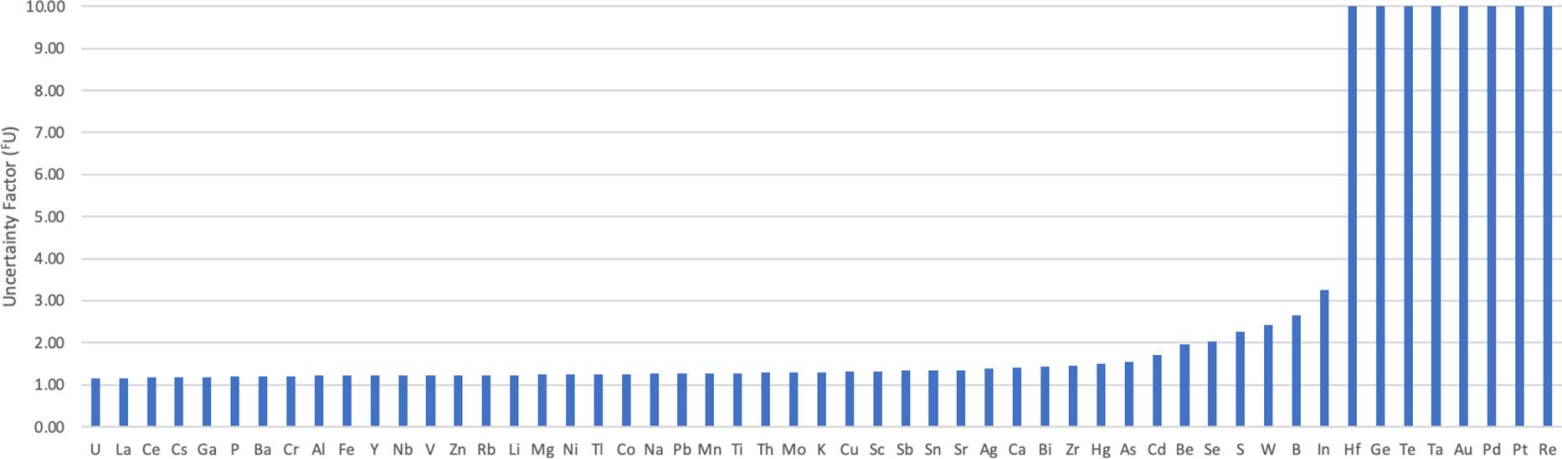
MU of measurement values (as ^*F*^*U*) for 53 elements determined by ICP in the GEMAS survey

The MU (including UfS) was also estimated as ^*F*^*U* for all 53 elements determined by ICP, as has been explained for As, excluding analytical bias as negligible in all cases (Fig. 6). One advantage of using ^*F*^*U* to express MU is that is applicable across all elements, from 1.01 for elements with very low MU (equivalent to *U’* of 1%) to the highest levels of MU (*U’* > 100%), whether their frequency distribution is normal or log-normal. Most of the elements studied (i.e. 27) have an ^*F*^*U* value of less than 1.3, which is approximately equivalent to an expanded *U’* of less than 30%. For 40 of the elements ^*F*^*U* is less than 2.0. Arsenic with an ^*F*^*U* of 1.54 is clearly intermediate in MU, but Pb with an ^*F*^*U* of 1.26 has a considerably lower MU. Eight elements (Hf, Ge, Te, Ta, Au, Pd, Pt, Re) have very large MU with estimated ^*F*^*U* value larger than 10 and are discussed further below in the context of FnFP.

## Fitness (FnFP) of measurements for the purpose of geochemical mapping for 53 elements

Although the level of MU varies widely between the 53 elements (^*F*^*U* from 1.01 to > 10), one derived judgement is whether the measurement procedure (sampling and analysis) is fit for the purpose of geochemical mapping. There are several different criteria for judging the FFP measurement results (and procedures) (Ramsey et al., 2019—Sect. 16), but the one criterion usually applied to geochemical mapping (and already mentioned, from Ramsey et al., 1992) does not depend solely on level of the MU, but on how it compares with the general (between-target) geochemical variability. If the geochemical variation is large across space, then consequently it does not require such a low level of MU to describe variation in a geochemical map.

As already discussed, the Fitness for Purpose (FnFP) criterion generally agreed for reliable geochemical mapping is that the sum of the sampling and analytical variance (i.e. the measurement variance) should not contribute more than 20% to Total Variance. This is the same as requiring that the geochemical variance should contribute more than 80% to the total variance. A consequence of this criterion is that one measurement method can be FFP for one area of high geochemical variability, but not FFP for a different area where the geochemical variability is much lower.

**Fig. 7:**
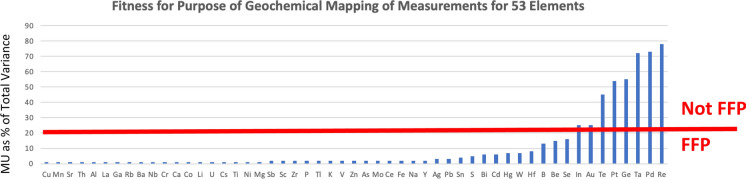
FnFP of measurements for Geochemical Mapping (for 53 elements), showing that the measurement values for only 8 elements (In, Au, Te, Pt, Ge, Ta, Pd, Re) are not generally fit for this purpose in the GEMAS agricultural soil survey across Europe, because MU contributes over 20% of the total variance

For the GEMAS geochemical mapping of agricultural top soils in Europe, the measurement procedure (Sampling + Analysis by ICP) is FFP for 45 out of 53 elements using this criterion (Fig. 7). For example, for As the MU contributes 2%, and for Pb 3%, to the total variance, which are well below the FFP criterion of 20% in this area. However, the measurement procedure is not FFP for 8 elements (In, Au, Te, Pt, Ge, Ta, Pd, Re), where the MU as a proportion of total variance is consistently over 20% (i.e. 25% to 78%, Fig. 7). This list of 8 elements is not quite that same as the list above for the elements that have ^*F*^*U* > 10, in that Indium has a ^*F*^*U* of 3.2 (i.e. considerably less than 10) but is still not FFP. Conversely, Hafnium has a ^*F*^*U* > 10, but the MU still only contributes 8.1% to the total variance, so is judged to be FFP (i.e. < 20%). This demonstrates the advantage of judging FFP, not by MU in isolation, but by MU as a function of the geochemical variation being described.

**Fig. 8:**
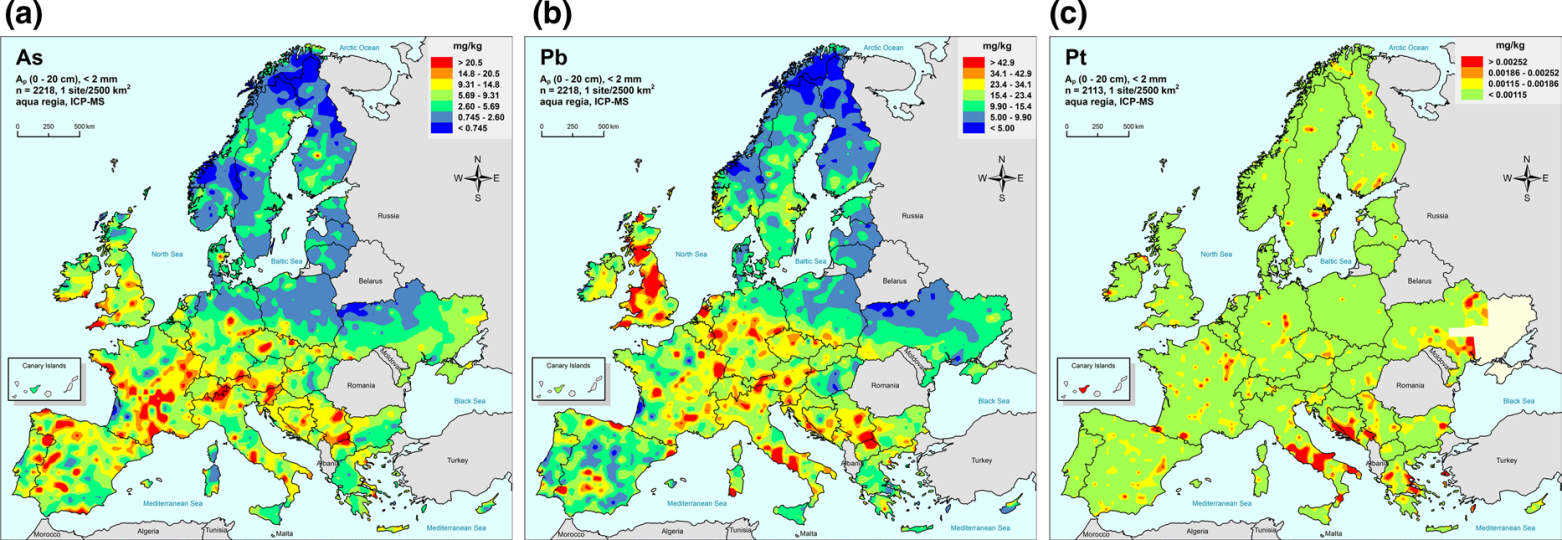
Geochemical maps of European agricultural top soils (GEMAS) for **a** As, **b** Pb and **c** Pt. (Reimann et al., 2014b), reproduced with permission. Map **c** demonstrates the lack of geochemical information in the lowest class interval for Pt concentration (< 0.00115 mg kg^−1^, shown in green), caused by the dominant effect of the measurement uncertainty that contributes 54% of the total variance (i.e. over the FnFP limit of 20%)

For Pt, discussed below, its MU contributes 54% to the total variance, which is well over the FnFP target of 20%. This measurement procedure is, therefore, not FFP for this area of Europe as a whole. The effects of this ratio can be seen in the resultant published maps for three elements: As, Pb and Pt (Reimann et al., 2014b)(Fig. 8).

The maps for As and Pb (Fig. 8a and b) both show clear delineation of the geochemical variability in areas of both low and high concentration. By contrast, for Pt (Fig. 8c) the areas of low concentration (< 0.00115 mg kg^−1^, shown in green) show no visible geochemical structure. Only for the areas of the highest Pt concentration (> 0 0.0018 mg kg^−1^, shown in red and orange) does there seem to be coherent hot-spots (e.g. in central Italy). Interestingly if only the 8 duplicates that were taken in these areas of highest Pt are used for the ANOVA, the value of MU as ^*F*^*U* falls from > 10 down to 2.15. However, the procedure still does not reach the FFP criterion in these areas, as MU still contributes 62% to the total variance. However, the MU for Pt in these high areas is dominated by sampling (56%), whereas for the whole area it is 100% dominated by the analytical procedure because of its detection limit (which is quoted as 0.0016 mg kg^−1^ and experimentally determined as 0.0007 mg kg^−1^ (Reimann et al., 2009)).

## Quantification of *in situ* analyte heterogeneity in European top soils using the duplicate method

One other benefit of using the duplicate method in geochemical mapping surveys is to estimate how heterogeneity of sampling targets varies at the large scale. This approach is discussed in more detail in a later section, but for the GEMAS study this heterogeneity is expressed as (^*F*^*u*_*samp*_) or (^*F*^*U*_*samp*_, for 95% confidence). This is the component of the MU, expressed as the uncertainty factor that arises from the sampling procedure.

**Fig. 9:**
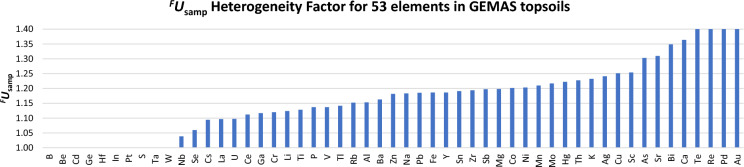
The variation of heterogeneity (as ^*F*^*U*_*samp*_) at the 10-20m scale between 53 elements in agricultural top soils surveyed in the GEMAS study

It is clear that there is an enormous variation apparent between different analytes in the degree of heterogeneity at this scale (10-20m) across Europe (Fig. 9). The estimated heterogeneity (as ^*F*^*U*_samp_) varies between apparent zero (^*F*^*U*_samp_ = 1) and high levels (^*F*^*U*_samp_ =  > 1.4). However, it must be appreciated that these are only estimated values. For example, Pt has an estimated ^*F*^*U*_samp_ of 1.00, but this is probably because nearly all of the measurement values are less than the detection limit, so that *U*_anal_ dominates the MU and *U*_samp_ is therefore effectively invisible.

Most elements (34) have lower levels of heterogeneity (^*F*^*U*_samp_ < 1.20), and a further 12 have intermediate levels (^*F*^*U*_samp_ 1.2 to 1.3). Seven elements have higher heterogeneity with estimated ^*F*^*U*_samp_ > 1.3 (Sr, Bi, Ca, Te, Re, Pd, Au). This high heterogeneity does not necessarily affect the FFP of the measurement (including sampling) procedure, as seen by the previous discussion of FnFP, e.g. for *As *^*F*^*U*_samp_ = *1.30.* These quantitative estimates of analyte heterogeneity at this scale may indicate other processes, such as the mode of deposition (see later discussion).

## Effect of MU on geochemical interpretation (e.g. As-ICPMS-GEMAS)

In addition to judging FnFP, a second use of MU values is to improve the reliability of geochemical interpretation that is based upon measurement values. One example that can be applied to the GEMAS measurement of As in top soils, is to decide if there are areas within Europe where the As concentration in topsoil exceeds a specified threshold value (T). For As this threshold has been set at 50 mg kg^−1^ for top soils with pH > 5 in the UK (UK Government, 2018), but many other elements in agricultural soils for Europe are covered more generally in EC Directive 86/278/EEC.

**Fig. 10:**
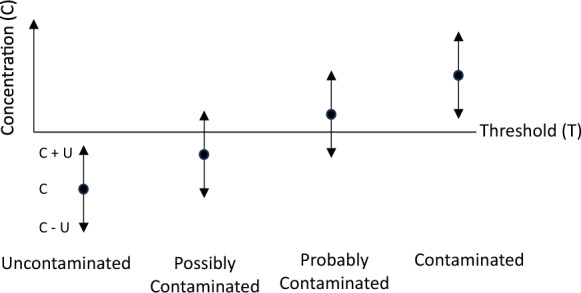
Effect of MU on compliance decisions against a regulatory threshold, enabling a probabilistic classification. In the case of As in GEMAS agricultural top soils, *T* = 50 mg kg^−1^, and ^*F*^*U* of 1.54, so an ‘uncontaminated’ soil would therefore need a measured As concentration value of < 32 mg kg^−1^ (i.e. when UCL of measured value of 32 is 50, which is 32* 1.54), rather than the < 50 mg kg^−1^ used in a deterministic classification that ignores MU

The general principle of how MU can be allowed for in the making of compliance decisions is by the use of a probabilistic approach (Ramsey & Argyraki, 1997, Williams and Magnusson, 2021) shown in Fig. 10. Only when the measurement value with the MU added (i.e. the UCL) is below the threshold (*x* + *U* < *T*) can the soil in the sampling target be said to be ‘uncontaminated’ with 95% confidence (i.e. 2.5% chance of true concentration being over T). Similarly, only when the measurement value minus the MU (I.e. the LCL) is above the threshold (*x* − *U* > *T*) can the sampling target be said to be ‘contaminated’ with 95% confidence (97.5% chance of true concentration being over T). For intermediate levels of contamination, we can also classify some targets as being either ‘possibly contamination’ (*x* < *T* < *x* + *U*, possibility of false positive classification) or ‘probably contaminated’ (*x* – *U* < *T* < *x*, possibility of false negative).

For the example of As by ICP-MS in the GEMAS topsoils, the MU is estimated as an Uncertainty Factor ^*F*^*U* of 1.54. Given *T* = 50 mg kg^−1^, we have the upper limit of a definitely ‘uncontaminated’ target at 32 mg kg^−1^ (at which the UCL is *x* x^*F*^ *U* = 32 × 1.54 = 50 mg kg^−1^). This figure of 32 mg kg^−1^ can also be calculated, for this particular data set, by applying the MU to the threshold, (i.e. T/^*F*^*U* = 50/1.54 = 32). Similarly, for the lower limit of the ‘definitely contaminated’, we have 77 mg kg^−1^ (at which the LCL is *x*/^*F*^*U* = 77/1.54 = 50 mg kg^−1^, or as *T*x^*F*^ *U* = 50 × 1.54 = 77 mg kg^−1^). Possibly contaminated targets will be in the range of measured values 32 to 50 mg kg^−1^, and ‘probably contaminated’ in the range 50 to 77 mg kg^−1^. It is then possible to use these four concentration ranges to make a probabilistic map that identify targets areas that are reliably in these four categories (Bettencourt et al., 2022). For example, ‘uncontaminated’ targets would use the threshold of < 32 mg kg^−1^, rather than the < 50 mg kg^−1^ that would be used in a deterministic classification that ignored MU.

## Method: MU Estimation at micro (μm) scale using oxygen isotope ratios in candidate reference materials (RMs)

The Duplicate Method can also be applied at the micron scale, and this has been demonstrated in a study of oxygen Isotopes ^18^O/^16^O (= ∂^18^O) in quartz candidate RMs using Secondary Ion Mass Spectrometry (SIMS) (Ramsey & Wiedenbeck, 2017). The purpose of the geochemical interpretation is this case is to minimise analyte heterogeneity within the candidate RM, which maximises performance of SIMS (e.g. repeatability of 0.01% = 0.1 ‰), by having the lowest possible MU that includes UfS. Incidentally, this purpose is almost the direct opposite of that used in the geochemical mapping example already discussed, which aimed to reveal the analyte heterogeneity between-targets (expressed as geochemical variance).

**Fig. 11:**
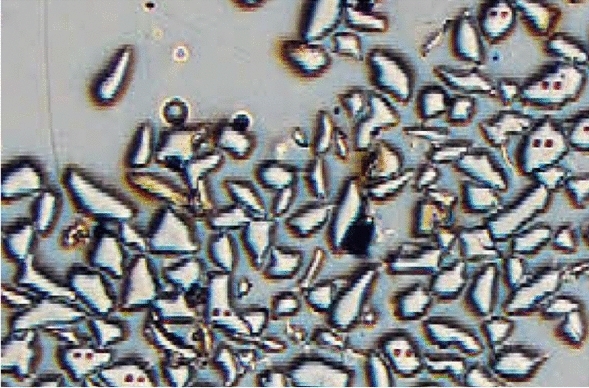
Fragments of candidate quartz RM NBS28 in plan view showing several of the 100 fragments where two craters (50 μm apart) where samples were taken for application of the Duplicate Method to estimate within- and between-fragment heterogeneity, and hence MU

For each of four candidate RMs, the heterogeneity of the isotope ratio ∂^18^O was estimated (as UfS) using the Duplicate Method applied to 100 sampling targets. A simplified balanced design was used, in which no analytical duplicates were made on either of the sample duplicates (Fig. 1, second variant). For one candidate quartz RM (NBS 28), each sampling target was one of the many component fragments (or grains) of quartz, each with a mean diameter of around 230 μm and mean mass estimated as approximately 20 μg. The duplicate samples/measurements were taken 50 μm apart, on the same fragment, leaving craters where a mass of 300–350 pg had been removed (Fig. 11). The spatial separation of 50 μm reflects that both crater locations were equally likely to have been selected to represent each fragment within this sampling procedure. The duplicate measurements were taken on 100 fragments, at different times in the analytical run selected at random, over a period of around 15 h. Machine drift was monitored and corrected retrospectively, where it was statistically significant. The variance components were estimated by applying classical ANOVA to the ~ 200 measurement values. Because a simplified experimental design was chosen, the purely analytical component of the variance was estimated independently using the instrument’s own software calculations based on repeated measurements.

## Results: estimates of heterogeneity and MU from the duplicate method applied at the micron scale

It is traditional practice in isotope geochemistry to report MU (and repeatability and heterogeneity) as relative standard uncertainty (i.e. *u’* at 68% confidence. k = 1), rather than expanded to 95%, and in parts per thousand (‰) called ‘per mil’, rather than in percent (%).

The repeatability of the measurements (sampling + analytical) estimated from ‘within-duplicate’ fragments was 0.14‰ (Table 2, row 2). This is somewhat larger than instrumental repeatability of 0.08 ‰ that was estimated outside the ANOVA using the instrumental software on replicating readings. There was therefore a small amount of heterogeneity within duplicate pairs, but this was much less than the heterogeneity between fragments (0.28 ‰) which was the main component of total variance = ‘total repeatability’ MU (0.31‰). When expressed as % of Total Variance, the variation between fragments (81%) was much larger than within-duplicate fragments (19%) (Table 3, row 3). For this application we ideally need minimal between-fragment variation (i.e. heterogeneity), say of < 0.3‰.

**Table 2:** Estimates of MU and its components made using classical ANOVA applied to δ^18^O on duplicated samples (‘dups’) in nearly 100 fragments of NBS28 quartz RM (Values extracted from Ramsey & Wiedenbeck, 2017)

	Total	Between-dups ~ ’geochemical’	Within-dups ~ ‘meas’	Instrumental ~ ‘analytical’	Number of pairs
Uncertainty as *u’* (‰)	0.31	0.28	0.14	0.081	97
% of Total variance	100	81	19	6.7	97

**Table 3:** Comparison of the repeatability components of MU for δ^18^O in ~ 100 fragments of four different candidate quartz RMs, made using classical ANOVA. High levels of between-fragment repeatability reveal a high level of analyte heterogeneity that is unacceptable in the case of one RM (GFZ-Qz1) (Values mainly extracted from Ramsey & Wiedenbeck, 2017)

Candidate Quartz RM	Between-fragment heterogeneity	Within-fragment repeatability
NBS28	0.28	0.14
GFZ-Qz1	2.30	0.10
ZRM1	0.18	0.16
MfN-Qz2	0.22	0.14

Similar experiments were undertaken of three other candidate quarts RMs in that study, and the results compared (Table 3).

The within-duplicate (within-fragment) repeatability is quite constant across the 4 candidate RMs (0.10 − 0.16 ‰, Table 3). However, the between-fragment MU (i.e. *u*_betn-fragment_ = heterogeneity between-fragments) is much more variable. To achieve the full performance of SIMS (e.g. 0.1 ‰) requires an RM with low heterogeneity, so FFP target is *u*_betn-fragment_ < 0.3 ‰. One RM (GFZ-Qz1) had the very high heterogeneity of 2.3‰ which indicates that this RM is not FFP. The other 3 Candidate RMs do meet this FFP criterion with their heterogeneity (between-fragment) being less than the target of 0.3 ‰.

The more general conclusion is that the Duplicate Method has been shown to be equally applicable for investigations at the micron scale. The fact that two different FFP criteria were applied in these studies, does not detract from the general applicability of this same experimental design at these very different spatial scales.

## Estimation of heterogeneity (as U_hetero_) using in situ measurements and applied over a range of spatial scales

### ***Method for heterogeneity estimation (as U***_***hetero***_***)***

The final application of the Duplicate Method illustrates three potential further uses that are considered in turn. One use is to estimate MU (random component) of in situ measurements (i.e. where no physical sample is removed from the sample target). A second use is to quantify analyte heterogeneity (U_hetero_), to see whether the in situ heterogeneity is diagnostic of the source of the contamination (briefly discussed above for the GEMAS study). Thirdly, estimates of U _hetero_ over a wide range of spatial scales, between μm and km, provide geochemists with a new source of geochemical information.

**Fig. 12:**
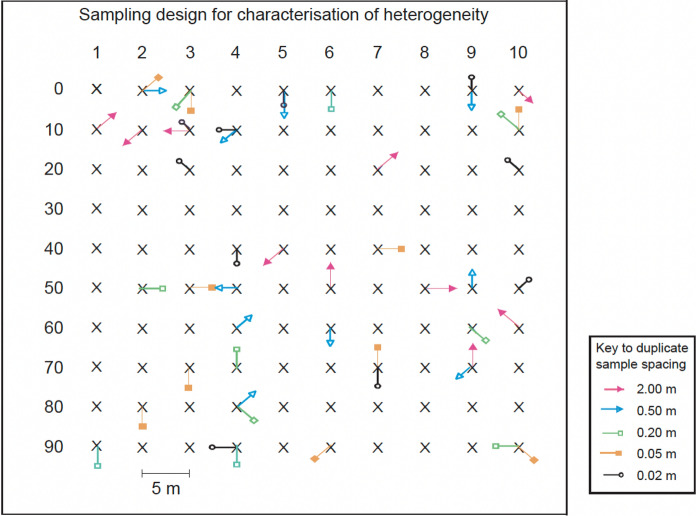
Sampling design quantification of heterogeneity over a range of seven spatial scales across a contaminated land site, using in situ PXRF. The key shows the spatial separation of the sample/measurement duplicates at each of the 100 sampling targets, indicated by an ‘X’, with a 5-m grid spacing. The arrows show ten locations, chosen at random, for duplicate sampling points at each sampling scale (with a different colour for each spatial scale) (Ramsey et al., 2013)

The equivalent of ‘duplicate samples’ to estimate the random component of MU for measurements, is taken by placing the in situ measurement device twice in a way that reflects two independent interpretations of measurement procedure. Usually, these two sampling points are both equally likely interpretations of the procedure, given that particular surveying technology. One example of this approach was used in an experiment to quantify analyte heterogeneity of Pb over range of scale one contaminated land site (50m by 50m) (Ramsey et al., 2013). In this study, duplicate measurements of Pb concentration were made using in situ portable X-ray fluorescence spectrometer (PXRF). Ten duplicate pairs of PXRF measurements were located at each of seven different distances apart (i.e. 0.02, 0.05, 0.2, 0.5, 2.0, 5.0 and 20 m apart) at 10 targets selected randomly from 100 targets on a regular grid across each of three sites (Fig. 12).

A simplified experimental design (i.e. Figure 1, second variant with no analytical duplicates) was used for speed at all but one scale (0.2m) where the full balanced design (Fig. 1 first option) was applied in order to estimate a value of *U*_anal_ that was applicable to all of these spatial scales. The heterogeneity (as *U*_hetero_) at each scale was estimated as the UfS (assuming that heterogeneity is the dominate source), by applying classical ANOVA to the 20 measurement values (log-transformed) for each of the seven spatial scales.

### ***Results of heterogeneity estimation (as U***_***hetero***_***)***

The frequency distribution of the Pb measurements at one site (Gang Mine, Derbyshire, UK) was generally log-normal, so the measurement values were log-transformed. The heterogeneity was expressed therefore as the standard ‘heterogeneity factor’ (HF), which is equivalent to the portion of the standard uncertainty factor that arises from just the sampling procedure (^*F*^*u*_*samp*_, k = 1).

**Fig. 13:**
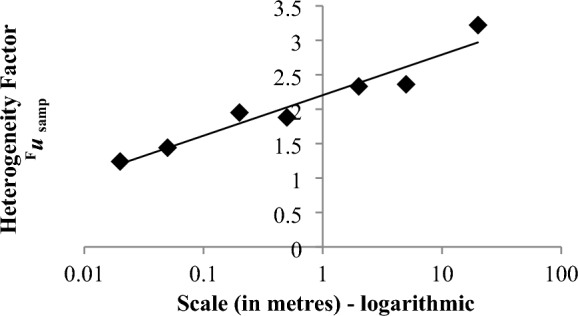
The variation in heterogeneity factor (^*F*^*u*_*samp*_, k = 1) with spatial scale (from 0.02 to 20 m) for Pb-contaminated land at Gang Mine (Derbyshire, UK) (revised from Ramsey et al., 2013)

The heterogeneity of the Pb concentration (expressed as ^*F*^*u*_*samp*_), clearly increases in a broadly linear fashion against the logarithm of the spatial scale at this site from around a value of around 1.2 to 3.2 (Fig. 13).

In order to investigate the wider implication of this heterogeneity quantification, this same experimental design was applied to two other sites in the UK (Stoke Bardolph in Nottinghamshire, and Coseley in Wolverhampton). In addition, the same HF statistic for approximately the scale of ~1m (i.e. 0.2 – 2m) was also retrieved from previous studies on ten other contaminated land sites with varying degrees of Pb contamination in their soil, arising from different types of source.

Heterogeneity factor of Pb at the approximately the same scale (~1m) can be seen to vary from 1.03 to 2.4 (*u’* approx. <5% to >100%) across these 12 sites (Table 4). The level of heterogeneity seems to be broadly diagnostic of the mode of deposition of Pb. For example, the heterogeneity factor is high (HF = ^*F*^*u*_samp_ = 1.4 – 2.4) for spatially uneven sources such as mine waste, canal dredgings, firing range and landfill. By contrast, the heterogeneity factor is lower (HF = ^*F*^*u*_samp_< 1.4 ) for more even sources such as sewage drying pans, flood plains >20km downstream of mines and smelter fume. It must also be recognised that the current heterogeneity of Pb may well have been affected by later remobilization (e.g. at low pH, or by subsequent ploughing).

**Table 4:** Increasing levels of Pb heterogeneity (within-target, expressed as standard heterogeneity factor HF = ^*F*^*u*_*samp*_) at the ~ 1m scale across 12 sites with different modes of Pb deposition

Sites	Location (within UK)	Heterogeneity (*HF* = ^*F*^*u*_*samp*_)
Sewage drying pans	Nottingham, Stoke Bardolph	**1.03**
Playing field-flood plain	Nottingham, R. Trent	**1.07**
Landfill now camp site	Littlehampton	**1.21**
Field near Pb smelter	Avonmouth	**1.25**
Pb smelting site	Wirksworth	**1.25**
Garden & allotment	SE London	**1.32**
Canal dredgings site	Coseley, West	**1.40**
Landfill	Hounslow Heath, East	**1.61**
Canal dredgings site	Coseley, East	**1.81**
Former Pb Mine	Black Rock	**1.97**
Former Pb Mine	Gang Mine	**2.33**
Ex-firing range	Hounslow Heath, West	**2.39**

The lower levels of the heterogeneity factor (HF < 1.4) tend to be associated with more even sources such as sewage drying pans, flood plains > 20 km downstream of mines and smelter fume. In contrast, the higher levels of heterogeneity (HF = 1.4 – 2.4) are associated with more spatially uneven sources such as mine waste, canal dredgings, firing range, and landfill. (Values and sources of original data sets cited, in Ramsey et al., 2013)

## Conclusions

It has been shown that knowing uncertainty of measurement values (MU) is crucial for their reliable geochemical interpretation. The estimation of MU, including the contribution from sampling (UfS), can be made using the Duplicate Method for both *ex situ* (i.e. lab measurement on extracted samples) and *in situ* measurements (made without extracting a physical sample). The duplicate method can use a full balance design, but also the less expensive unbalanced or simplified design, and is applicable to any sampling medium: soil, sediment, herbage, waters, gases, *etc..* The duplicate method can also be applied at any spatial scale, from the micro to the macro. At the µm scale, it can be applied to the estimation of δ^18^O in quartz grains by SIMS, when in that example low geochemical (between-target) variance is required. In contrast, at the km scale for the geochemical map of Europe, the Duplicate Method can be used to show whether the high proportion of geochemical variance required for reliable maps has been achieved (using a fitness for purpose criterion of MU being less than 20% of the total variance). The uncertainty factor for 53 elements measured in the GEMAS survey of agricultural topsoils across Europe varied from 1.01 to >10. These different levels of MU have implication for the geochemical interpretation of the measurement values made for decisions such as unacceptable levels of contamination, and enable a probabilistic, rather than deterministic, approach to mapping and classification.

The Duplicate Method has also been applied to quantify the analyte heterogeneity using an *in situ* measurement device (i.e. PRXF), across a range of spatial scales. The heterogeneity can be expressed as a heterogeneity factor (which can be quantified as the part of the uncertainty factor that is due to sampling).

MU values can therefore be useful to:- (1) improve geochemical interpretation, such comparing when geochemical measurement values to decide whether they are really different (in space or time) by carry data quality into the interpretation (expressed as MU), (2) show whether measurement results and procedures (including sampling) are Fit for Purpose (FFP), such as classification of material against a threshold or for geochemical mapping, and (3) decide whether either better analysis, or better sampling, is required to make sufficiently reliable geochemical decisions.
